# Evolutionary forces shaping genomic islands of population differentiation in humans

**DOI:** 10.1186/1471-2164-13-107

**Published:** 2012-03-22

**Authors:** Tamara Hofer, Matthieu Foll, Laurent Excoffier

**Affiliations:** 1 Computational and Molecular Population Genetics Lab, Institute of Ecology and Evolution, University of Bern, 3012 Bern, Switzerland; 2 Swiss Institute of Bioinformatics, 1015 Lausanne, Switzerland

## Abstract

**Background:**

Levels of differentiation among populations depend both on demographic and selective factors: genetic drift and local adaptation increase population differentiation, which is eroded by gene flow and balancing selection. We describe here the genomic distribution and the properties of genomic regions with unusually high and low levels of population differentiation in humans to assess the influence of selective and neutral processes on human genetic structure.

**Methods:**

Individual SNPs of the Human Genome Diversity Panel (HGDP) showing significantly high or low levels of population differentiation were detected under a hierarchical-island model (HIM). A Hidden Markov Model allowed us to detect genomic regions or islands of high or low population differentiation.

**Results:**

Under the HIM, only 1.5% of all SNPs are significant at the 1% level, but their genomic spatial distribution is significantly non-random. We find evidence that local adaptation shaped high-differentiation islands, as they are enriched for non-synonymous SNPs and overlap with previously identified candidate regions for positive selection. Moreover there is a negative relationship between the size of islands and recombination rate, which is stronger for islands overlapping with genes. Gene ontology analysis supports the role of diet as a major selective pressure in those highly differentiated islands. Low-differentiation islands are also enriched for non-synonymous SNPs, and contain an overly high proportion of genes belonging to the 'Oncogenesis' biological process.

**Conclusions:**

Even though selection seems to be acting in shaping islands of high population differentiation, neutral demographic processes might have promoted the appearance of some genomic islands since i) as much as 20% of islands are in non-genic regions ii) these non-genic islands are on average two times shorter than genic islands, suggesting a more rapid erosion by recombination, and iii) most loci are strongly differentiated between Africans and non-Africans, a result consistent with known human demographic history.

## Background

A number of studies investigated patterns of divergence between closely related, sympatric species and identified so-called 'islands of speciation' in the genome, where the divergence was particularly strong [1-3]. These highly differentiated genomic islands might emerge due to divergent selection acting on the two species [4,5]. Divergent genomic regions cannot only be observed between species, but also between populations within species. Humans are the least differentiated of the extant primate species [6] and most genetic variation is found in populations rather than between populations [7]. Levels of population differentiation are determined both by demographic factors such as genetic drift and gene flow, which can increase or respectively decrease population differentiation [8], and by selective processes, which can also promote [e.g. [9]] or lower [e.g. [10]] differentiation [11-13]. While it is usually believed that demographic forces globally act on the whole genome, it has been shown that pure neutral processes can affect allele frequencies at specific loci during range expansions [14,15], and increase allele frequencies in newly colonised areas [16-18]. This allelic surfing phenomenon depends on local demographic patterns [16] but can be also be enhanced by spatial bottlenecks [19,20].

In the genomics era, large-scale human datasets such as the HapMap project [21-23], the Human Genome Diversity Panel [HGDP; [7,24]], or recently the 1,000 Genomes project [25] can be analysed to reconstruct the demographic history of populations or to find signatures of selection on the genome. Rosenberg *et al. *[26] investigated the genetic relationship of human populations and found that populations from the same continent share more ancestry than random populations [see also [7]]. Coop et al. [27] found most evidence for selection between continental groups suggesting that these are ancient adaptations that potentially occurred during the colonisation of continents. There are rather few examples for very local patterns of selection in humans [see e.g. [28-31]]. Recently, Hernandez *et al. *[32] argued that reduced diversity and increased population differentiation in exons could partly result from background selection rather than from selective sweeps, but alternative forms of adaptation, such as selection on standing variation or on multiple beneficial alleles could contribute to population differentiation [see e.g. [33,34]].

In this study we aimed at identifying regions in the human genome with elevated or decreased levels of population differentiation potentially indicative of past episodes of selection. We used the HGDP-CEPH Human Genome Diversity Panel [7] including 660,664 SNPs typed in 53 populations to reliably infer population differentiation. Indeed, whereas the HapMap [21-23] or the 1,000 genome [25] panels provide more detailed genomic information than the HGDP SNP panel, they have been tested in far fewer populations making them actually less powerful for detecting outlier SNPs. We used a novel method to identify loci with unusual *F_ST _*values that takes into account hierarchical structure of human populations [35]. Using the significance of individual SNP *F_ST _*values as observations, we used a HMM to identify genomic regions with average, high or low population differentiation hidden states. Based on a large set of populations, we thus provide an extensive map of significantly differentiated genomic islands, whose properties such as size, location in the genome, recombination rates or overlap with genic regions, were assessed to collect evidence for the respective effects of selection and neutral demographic processes.

## Results

### Selection test

**Figure 1:**
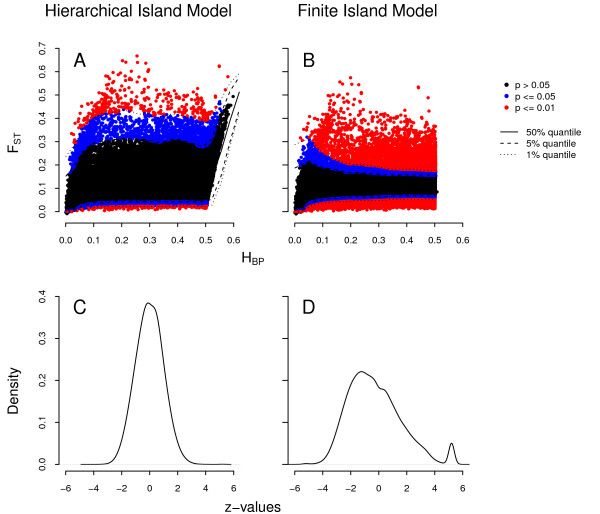
**Detection of outlier SNPs based on *F_ST _***. Joint distributions of *F_ST _*and *H_BP _*for SNPs on chromosome 4 (A, B) and genome-wide distribution of SNP *z*-values (C, D). Locus specific *p*-values and *z*-values were computed either based on the hierarchical island model (A, C) or the finite island model (B, D). Chromosome 4 is representative of the joint distribution of these statistics on all other chromosomes.

By simulating the joint null distribution of *F_ST _*and heterozygosity between populations (*H_BP_*) under both a Finite Island Model (FIM) [36] and a Hierarchical Island model (HIM) [35], we obtained the significance of SNP-specific *F_ST_*s under these two models (Figure 1). We find as many as 21.80% of all SNPs with significant *p*-values at the 1% level under a FIM, while this proportion reduces to 1.5% under a HIM. The excess of significant loci observed under the FIM likely includes many false positives, due to the unrealistic assumption of even levels of differentiation between all pairs of populations [35]. Indeed, the *F_ST _*distribution simulated under the HIM much better fits the observed distribution of *F_ST _*values than that simulated under the FIM (see Additional file 1). We thus do not find much evidence of selection under the HIM, but neither *F_ST _*nor their associated *p*-values are randomly distributed along chromosomes (run-tests *p*-value < 0.001 for all chromosomes). It implies that there are genomic regions with generally elevated or decreased levels of population differentiation, which we have tried to identify using a model-free HMM approach. To this aim, *F_ST _p*-values were first transformed into *z*-values, also called normal scores, as observations for the HMM. *Z*-values are expected to follow a normal distribution under a proper null model, and interestingly, this is only true for *z*-values obtained under the HIM, while *z*-values computed under the FIM have a right-skewed distribution (Figure 1). The shape of these distributions thus gives us further evidence that the HIM better fits the data than a FIM, since we expect that most SNPs are actually neutral. Given the unrealistic assumption of the FIM and its associated non-Normal distribution of the *z*-values, all results presented below will be based on the HIM only.

### Genomic islands

We used a two-step HMM approach to identify regions in the genome with significant population differentiation than average (see Methods and Additional file 2). We first broadly defined genomic regions with generally increased or decreased population differentiation using standard HMM algorithms. In a second step we controlled the False Discovery Rate (FDR) of SNPs and retained only those regions that contained at least one SNP with a genome-wide FDR of 0.001 (hereafter called FDR SNPs). Hereafter, we shall call *high-differentiation islands *(HDIs) those genomic regions with significantly high levels of population differentiation, and *low-differentiation island*s (LDIs) those regions with significant low levels of population differentiation.

**Figure 2:**
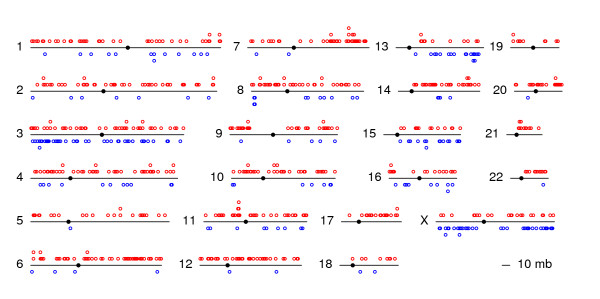
**Distribution of HDIs and LDIs**. Genomic distribution of HDIs (red) and LDIs (blue). Chromosomes are represented by lines with a filled circle at the centromere. Each circle represents a significant genomic island, as identified by a Hidden Markov Model.

Under the HIM we detected 625 HDIs as well as 197 LDIs (Table 1, Figure 2, and Additional file 3).

**Table 1:** Properties of genomic islands.

	High-differentiation islands (HDIs)		Low-differentiation islands (LDIs)	
	**Mean**	**Min-max**	**Mean**	**Min-max**

No. of islands	625		197	

Length (bp)	465,756	11,037 - 15,210,531	368,902	25,709 - 2,021,127

No. of SNPs	73.61	4 - 322	90.76	10 - 430

No. of genes	5.35	0 - 92	3.45	0 - 37

Mean Recombination rate	1.04	0.00 - 6.31	1.31	0.04 - 7.48

Mean F_ST_	0.23	0.17 - 0.43	0.09	0.06 - 0.14

Mean heterozygosity	0.23	0.09 - 0.33	0.31	0.18 - 0.42

Mean *z*-value	0.88	0.32 - 2.63	-0.77	-1.41 - -0.32

Properties of islands with significant levels of population differentiation as detected by the HMM approach

We find that the number of HDIs per chromosomes correlates with the number of SNPs (R = 0.859, *p*-value < 0.001), but the number of LDIs does not (R = 0.226, *p*-value = 0.301). Chromosomes 3 and X appear to have a higher density of regions with LDIs than the other chromosomes (see Figure 2), which, for the X chromosome, is not due to the overall higher observed level of differentiation since our HIM test controls for that (see below). The average heterozygosity in HDIs (0.23) is significantly below the genome-wide average of 0.28 (*t*-test, *p*-value < 0.001), whereas LDIs present a significantly higher heterozygosity of 0.31 (*t*-test, *p*-value < 0.001).

Previous outlier detection studies sometimes refrained from simultaneously analysing the autosomes and the X-chromosome at once, since demographic histories between autosomal and sex-chromosomes differ [37]. In our case, each chromosome was analysed separately, and individual SNP loci were analysed based on their transformed *p*-value instead of their absolute *F_ST _*value, allowing for a comparison of chromosomes with different average *F_ST_*. Note however that the largest HDI is located on the X-chromosome, in the low-recombination centromeric region between positions 55.9 and 67.0 Mb.

**Figure 3:**
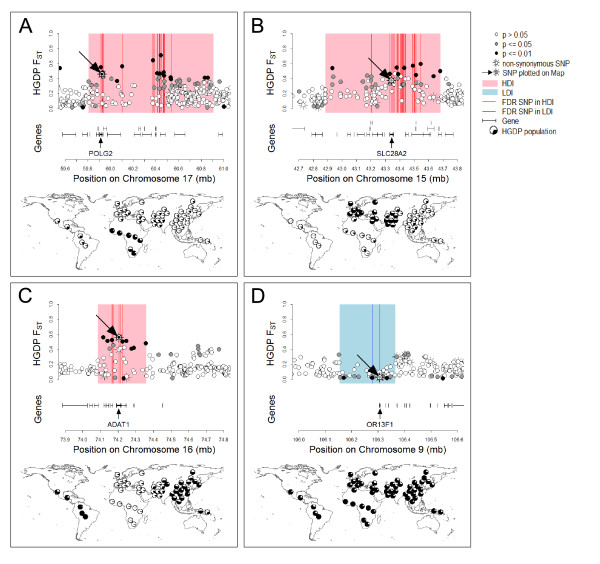
**Examples of genomic islands**. Each panel shows a genomic island that includes a significantly differentiated non-synonymous SNP. On top, the HDI or LDI is shown in details with all the SNPs and genes located in that region. SNPs are coloured according to their significance level in the selection test and FDR-SNPs are indicated by red vertical lines. The oblique arrow points at the non-synonymous SNP, and the vertical arrow indicates the gene embedding this SNP. The allele frequencies of the non-synonymous SNP in the HGDP populations are shown on the map below. Most common patterns of significant differentiation are found between continental groups, such as African vs. non-Africans (A), between Eurasian vs. non-Eurasians (B), or between East Asian and American vs. African and Eurasian populations (C). SNPs in low-differentiation islands tend to have similar allele frequencies in all populations (D).

**Figure 4:**
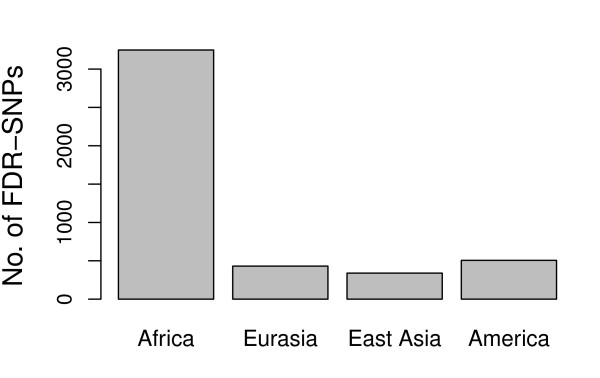
**Continental groups showing the largest degree of genetic differentiation**. For each FDR-SNP, we determined the allele frequency difference between populations in each continental group (Africa, Eurasia, East-Asia and America) and the rest of the world, and we assigned it to the continental group that showed the highest frequency difference. The histogram reports the number of FDR-SNPs in HDIs assigned to each group.

FDR SNPs in HDIs are found mainly differentiated between Africans vs. non-Africans (71.8%), and less between Americans vs. non-Americans (11.2%), Eurasians vs. non-Eurasians (9.6%), or East Asians vs. non-East Asians (7.5%; see Figures 3 and 4). More precisely, many FDR SNPs have high ancestral frequencies in Africa and low frequencies everywhere else.

Finally, it is worth noting that at odds with genomic islands results, the analysis of individual SNPs with significant population differentiation do not show any enrichment for genic or non-synonymous SNPs, leaving us with no direct evidence for selection at this level.

### Genic regions

We find that HDIs are enriched for genic regions. Indeed, 81.3% of high-differentiation regions overlap with at least one gene, which is significantly more than expected based on the size and the number of regions (*p*-value = 0.033). Contrastingly, LDIs are not enriched for genic regions, even though 76.6% of them overlap with at least one gene (*p*-value = 0.630). In keeping with these results, HDIs are significantly enriched for genic SNPs (*p*-value < 0.001), while LDIs are significantly depleted for genic SNPs (*p*-value < 0.001; Table 2 and Additional file 4). Interestingly, both HDIs and LDIs are significantly enriched for non-synonymous SNPs, which have a slightly significant higher *F_ST _*than other SNPs in HDIs (*p*-value = 0.030), and a significantly lower average *F_ST _*than other SNPs in LDIs (*p*-value = 0.004). We define here non-genic islands as those that do not overlap with any genic region (18.7% of HDIs and 23.4% of LDIs). The average distance of non-genic islands to the closest gene is less than 200 kb, which is not closer than expected (permutation test, *p*-value > 0.1 for both HDIs and LDIs). Remarkably, all LDIs as well as all but 4 HDIs overlap with transcription factor binding sites, which is significantly more than expected by chance for HDIs (permutation test, *p*-value = 0.002), but not for LDIs (*p*-value = 0.073), suggesting that non-genic HDIs might still be under some functional constraints.

**Table 2:** Enrichment of genic and non-synonymous SNPs in genomic islands.

SNP subset		Genic	Non-synonymous
High population differentiation	p-value(selection test) < = 0.01^a^	0.6576	0.1307
	
	Most likely states (Viterbi)^b^	< 0.0001^+^	0.0002^+^
	
	FDR < = 0.001^c^	< 0.0001^+^	0.0374^+^
	
	Islands^d^	< 0.0001^+^	0.0053^+^

Low population differentiation	p-value(selection test) < = 0.01^a^	0.2143	0.6402
	
	Most likely states (Viterbi)^b^	0.0303^-^	0.5888
	
	FDR < = 0.001^c^	0.0007^-^	0.6543
	
	Islands^d^	< 0.0001^-^	0.0083^+^

Fisher exact test for enrichment or depletion of genic and non-synonymous SNPs in genomic islands defined by the selection test or by the HMM approach

^a ^subset of SNPs that are significant in the selection test at the 1% level

^b ^subset of SNPs that belong to the high-differentiation or low-differentiation state as identified by the Viterbi algorithm in the first step of the HMM approach

^c ^subset of SNPs that are assigned to the high-differentiation or low-differentiation state under a genome-wide FDR = 0.001 in the second step of the HMM approach

^d ^subset of SNPs that are located within HDIs or LDIs

^+ ^enrichment of SNP category in SNP class

^- ^under-representation of SNP category in SNP class

We find that both HDIs and LDIs have a significant lower recombination rate than the rest of the genome (weighted *t*-test *p*-value < 0.001 for HDIs and *p*-value = 0.039 for LDIs). Since we find that the average size of non-genic islands is only about half the size of genic islands, we used an ancova to determine the association between the size of islands and local recombination rate, controlling for their genic or non-genic state. We find that HDI size is negatively related to recombination rate in genic HDIs (test of slope *p*-value < 0.001; Additional file 5) as well as in non-genic HDIs (*p*-value = 0.021). However, the regression slope is significantly steeper in non-genic than in genic HDIs (ancova test of slope difference *p*-value = 0.017), which suggests that recombination is more efficient in eroding HDIs in non-genic regions, potentially due to an absence of selective constraints in these regions. Contrastingly, island sizes are negatively correlated with recombination rate in genic LDIs (*p*-value < 0.001), but not in non-genic LDIs (*p*-value = 0.34) and the regression slopes of genic and non-genic LDIs are not significantly different (*p*-value = 0.077). Note also that HDIs overlap less with recombination hotspots than expected by chance (*p*-value < 0.001), unlike LDIs (*p*-value = 0.192). There are hotspots separating 9 out of 35 pairs of HDIs that are within 200 kb of each other, supporting the view that the HMM detected distinct signals of selection close to each other. We then tested for an enrichment or depletion of genes that overlap with HDIs or LDIs in biological processes using the PANTHER gene ontology database [38,39]. After Bonferroni correction, only two biological processes ('Lipid and fatty acid binding', *p*-value = 0.006; 'Glycogen metabolism', *p*-value = 0.046) are enriched for genes in HDIs, and a single process ('Oncogenesis', *p*-value = 0.005) is enriched for genes located in LDIs.

## Discussion

### A limited role for selection in humans

Using a hierarchical island model (HIM) to describe patterns of differentiation within and between human continental groups, we identified SNPs from the HGDP panel that presented unusual levels of population differentiation. The slight excess (< 1%) of significant SNPs under the HIM suggests a limited role for adaptive or balancing selection in the human genome, in agreement with a recent analysis of the 1,000 genome project [32]. Note that this excess could also be due to our inability to take into account the exact details of past human history [40], but it is important to underline that outlier loci are not randomly distributed along chromosomes, which motivated us to use a HMM to detect islands of high or low differentiation (HDIs and LDIs respectively). The fact that the joint distribution of *F_ST _*and *H_BP _*generated under the HIM much better fits the observed distribution than that obtained under the FIM (Figure 1 and Additional file 1) suggests that the HIM captures key aspects of human demography and that the identified outlier loci are more likely to be enriched for true signal of selection. The choice of the right demographic model thus appears crucial for the proper identification of selection signals.

### The action of neutral and selective processes in the genome

Several features of outlier SNPs and differentiation islands point towards the action of selection: i) HDIs are enriched for both genic and non-synonymous SNPs, ii) non-synonymous SNPs are more differentiated than other SNPs within HDIs, consistent with directional selection, and non-synonymous SNPs are less differentiated than synonymous SNPs within LDIs, consistent with purifying selection (see Additional file 6 for a list of non-synonymous SNPs with significant differentiation located in HDIs or LDIs), iii) non-synonymous SNPs are enriched in LDIs, compatible with balancing selection at a few SNPs and the accumulation of neutral mutations at other nearby sites, iv) LDIs are generally smaller than HDIs, in agreement with balancing selection giving more time for recombination to erode LDIs and fast selective sweeps creating large HDIs, v) the negative correlation between HDI size and recombination rate is stronger for genic than for non-genic HDIs, consistent with recent positive selection on genes and vi) HDIs overlap more often with genes than expected by chance, and there is a significant excess of transcription factor binding sites in HDIs. Note, however, that the HMM has more power to highlight regions with low recombination rates (such as functional regions), where neighbouring SNPs are more likely to have similar *F_ST _*values (but see Additional file 7).

Some other features of the islands are better explained by past demography than by selection, like i) levels of heterozygosity in LDIs that are comparable with genome-wide levels, unlike what would be predicted by balancing or background selection, ii) most high-*F_ST _*SNPs with low FDR that are mainly differentiated between Africa and non-Africa, and between America and non-America, which is compatible with the action of surfing after spatial bottlenecks (Figure 4), or iii) 23.4% of LDIs and 18.6% of HDIs do not overlap with any annotated functional gene. Interestingly, these non-genic islands (both HDIs and LDIs) are about two times shorter than islands overlapping with genes, which is consistent with the assumption that they are neutral and therefore more easily eroded by recombination. But note that most of the non-genic islands overlap with transcription factor binding sites, which are, however, less constrained than genic regions [41].

It thus appears likely that genomic islands with unusual levels of differentiation have been shaped both demographic and selective events, which still appear very challenging to disentangle without a higher density of markers and populations.

### Recently selected biological processes

We find evidence for local adaptation to food sources and nutrition as the biological processes of 'Lipid and fatty acid binding' and 'Glycogen metabolism' are enriched for genes located in HDIs. This result is in line with earlier studies showing that diet differs between populations and should present strong selective pressures [42,43]. Interestingly the process of 'Oncogenesis' is enriched for genes in LDIs. This process includes genes that normally regulate cell growth and differentiation [44] and cancer/testis genes that seem to be under rapid diversifying selection between human and chimpanzees, especially on the X-chromosome [45]. The X-chromosome is enriched for LDIs, supporting the interpretation that cancer/testis genes are under diversifying selection in humans, leading to balanced polymorphisms. On the other hand, we might have more power for the detection of LDIs on the X-chromosome due to its higher level of differentiation as compared to the autosomes. However, chromosome 3 has a level of population differentiation that is comparable to that of the other autosomes, but is also enriched for LDIs. A literature search did not reveal any neutral explanation for the high prevalence of LDIs on chromosome 3 leaving the possibility that chromosome 3 is enriched for targets of balancing selection.

### Comparison with other genome scan studies

HDIs contain several candidate genes for local adaptation identified in previous studies, such as *TRPV6 *[46], *ASPM *[47], *prodynorphin *[PDYN; [48]], the *duffy blood group *locus involved in malaria resistance [DARC; [49]], or the *ectodysplasin A receptor *[EDAR; [50]]. Additionally, HDIs overlap with genes linked to skin pigmentation including the *melanocortin 1 receptor *gene [MC1R; [51]], *KITLG *[52], *SLC24A5 *[53], *tyrosinase-related protein 1 *gene [TYRP1; [54]], and *OCA2 *[55]. LDIs also overlap with genes that were previously shown to be under balancing selection such as *HLA-C *[56] and *dystrophin *[DMD; [57]]. While HDIs are more sensitive towards local adaptation, LDIs may fail to highlight genes that are under balancing selection in only a few populations. More generally, we tested the overlap of HDIs and LDIs with regions identified as being under positive and balancing selection in previous genome scans (Additional file 8). We actually find a clear overlap between HDIs and regions under positive selection detected with methods based on levels of population subdivision [58-60], analyses of the site frequency spectrum [61-64], or tests relying on patterns of LD and haplotype variability [23,28,65-67]. Interestingly, HDIs do not overlap with studies that aimed at detecting old episodes of selection based on the ratio of polymorphism relative to divergence among species [68-70], which suggests that we detect genomic islands that have appeared more recently, after the out-of-Africa event 50-60 Ky ago [13]. However, the fact that some genomic regions are detected with several selection tests does not really demonstrate the action of selection, but rather that the identified signal is strong enough to be picked up by various methods. It is also worth noting that several candidate regions for balancing selection identified in previous studies did not overlap with LDIs (Additional file 8), potentially because balancing selection events are rare in the human genome and very hard to detect [71,72], in agreement with the fact that we detect less LDIs than HDIs. The difficulty to detect balancing selection is further illustrated by the low concordance between the 5 previous studies aiming at detecting balancing selection, where only 1.3% of all identified candidate regions were detected in more than one study.

Future HMM approaches could be extended to the analysis of next-generation sequencing data instead of a limited number of linked markers. With deeper coverage and the inclusion of additional populations allowing precise estimation of levels of population differentiation, the 1,000 Genomes project [25] could provide a unique and very powerful tool to refine the delineation of islands of differentiation.

## Conclusions

The proper detection of loci with unusual levels of population differentiation requires an appropriate model of human genetic structure, such as the HIM model used here. While we find little direct evidence of selection at the level of individual SNPs, the identification of genomic islands of differentiation under an HMM approach pooling information over linked SNPs reveals more powerful. We find that several properties of genomic islands overlapping with gene regions are difficult to explain without the action of selection, but that past demographic events such as gene surfing are probably involved in their occurrence in non-genic regions.

## Methods

### Data

We analysed the HGDP-CEPH Human Genome Diversity Panel including a total of 660,918 SNPs typed in 53 populations worldwide [[7]; ftp://ftp.cephb.fr/hgdp_supp1/]. For subsequent hierarchical analyses, the 53 populations were grouped into the 5 major geographic regions defined by Rosenberg *et al. *[7,26]: Africa, Eurasia, East Asia, Oceania, and America. We removed 12 SNPs that have only missing data, 4 SNPs that were not typed at all in a population, 50 SNPs that were monomorphic in all populations, and we discarded 188 SNPs that were located on the Y-chromosome, on the pseudoautosomal region of the X and Y chromosome, or on mitochondrial DNA, leaving us with 660,664 SNPs for subsequent analyses.

### Selection test

We used the hierarchical selection test [35] implemented in ARLEQUIN ver 3.5 [73] to identify loci with significant levels of population differentiation. For each chromosome, ARLEQUIN generated the joint null distribution of global *F_ST _*[74,75] and heterozygosity between populations (*H_BP_*) based on 50,000 coalescent simulations under a hierarchical island model (HIM) or under a finite island model (FIM). Beaumont and Nichols [36] proposed to simulate the joint distribution of *F_ST _*and heterozygosity under a FIM, which they assumed to be robust under a wide range of conditions. However, Excoffier et al. [35] recently showed that the presence of hierarchical structure among sampled populations leads to an excess of false positives if the data is analysed under the assumption of a FIM. Instead the underlying continental structure of human populations [7,26] needs to be taken into account, which can be done by using a HIM [76]. In our study the simulated HIM consisted in 10 groups of 100 demes and the FIM of a single group of 100 demes. Migrations rates within and between groups were estimated from the observed F-statistics (Excoffier et al. 2009). Obtained null distributions were used to compute *p*-values for the individual SNPs and corresponding *z*-values (i.e. standard scores) with the quantile function *qnorm *implemented in the statistical software R [77]. For instance, a positive *z*-value of 1.64 corresponds to a *p*-value of 0.05 for high population differentiation whereas a *z*-value of -1.64 indicates a *p*-value of 0.05 for low differentiation. We used a run test [78] to test for a non-random distribution of *F_ST _*and *p*-values along chromosomes. Note that previous studies using outlier approaches sometimes restricted their analyses to autosomes because the differentiation level of the X-chromosome is higher due to its reduced effective size [e.g. [27,79,80]]. However, our model-based approach allows us to take into account the specificities of both autosomes and sex chromosomes and to compare them in the same analysis.

### Hidden Markov model

Sliding window approaches have often been used to find clustered values of some statistic along a sequence [see e.g. [63,81]], but this approach has some drawbacks. Indeed, the choice of the correct window size and increment is not trivial, and it might have a strong impact on the number and size of detected clusters. Additionally, random fluctuations of the test statistic in a delimited window might lead to the detection of a cluster when there is none [82]. Hidden Markov Models (HMM) are widely used in biology for sequence analyses, since they explicitly model dependencies among neighbouring markers [83-85] and they have recently been introduced to identify genomic regions influenced by selection [e.g. [86,87]]. To define HDIs and LDIs, we thus applied a HMM as implemented in the R package 'HiddenMarkov' [88]. We used a HMM with 3 hidden states for low (LDI), intermediate, and high (HDI) levels of population differentiation, respectively. A HMM is characterised by different parameters, such as the distribution of the test statistic under each state, emission probabilities, and transition probabilities. We used *z*-values as the observed test statistic for the HMM. The distribution of *z*-values under each of the 3 states was assumed to be Gaussian with a given mean and standard deviation estimated from the data. Emission probabilities specifying how likely it is to observe a given value under each state, and the transition matrix defining how likely it is to pass from one state into another state were also estimated from the data. We imposed a constrained transition matrix disallowing direct transitions between HDI and LDI states. The Baum-Welch algorithm [89] was used to estimate the parameters for the HMM for each chromosome independently. The algorithm was launched 1,000 times from different starting values and the resulting parameter estimates with the highest likelihood were retained for the final model (Additional file 9). Note that when we tried using a model with an unconstrained transition matrix, the transition probabilities between these two states were in all cases below 0.07.

We used a two-step approach to identify HDIs and LDIs (see Additional file 2 for an illustration of the method). In a first step the Viterbi algorithm [90] was used to determine the most likely sequence of states on a given chromosome and to define regions with different levels of population differentiation. We then identified SNPs that were significantly assigned to either the high-differentiation state or the low-differentiation state by computing their Local Index of Significance [LIS; [91,92]], which is the probability estimated from the HMM that this SNP does not belong to a given state. LIS takes into account linkage between neighbouring loci since it is based on the local dependence structure of the HMM. Following the approach of Wei *et al. *[92] we used the LIS to identify SNPs that were significantly associated with either high- or low-differentiation under a genome-wide False Discovery Rate (FDR) of 0.001 and that are called here FDR-SNPs. These FDR-SNPs are not necessarily the most significant SNPs as detected from the selection test alone, but they are rather located in regions with globally high or low levels of population differentiation.

Finally, we combined the output of the Viterbi algorithm and the FDR procedure by only retaining those regions that contained at least one high- or low-differentiation FDR-SNP to define the most significant HDI and LDIs, respectively.

### Recombination rate and recombination hotspots

We used standardized, sex-averaged DeCode recombination rates [93] to assess the impact of recombination on genomic islands of high or low population differentiation on the autosomes. We applied a weighted *t*-test [94] to test for differences in recombination rates between presumably neutral regions, HDIs and LDIs, respectively. Following Kong *et al. *[93] we defined recombination hotspots as those regions on the recombination map that have a standardized recombination rate greater than 10. We then determined the number of HDIs and LDIs that overlap with one or more recombination hotspots. To determine the significance of this value we randomly permuted islands 10,000 times across the whole genome.

### Functional analyses

Functional genes as annotated in ENSEMBL 54 [95] overlapping with HDIs and LDIs were identified. We determined the number of HDIs and LDIs that overlap with at least one gene (i.e. genic islands) and tested the significance of this value using 10,000 random permutations of islands across the whole genome. In this procedure, positions of HDIs and LDIs and regions in-between were permuted simultaneously to compute the null distribution of the overlap between islands and genes. We used an ancova model to test for an association between the length of islands and their average recombination rate, controlling for their genic or non-genic status. In this analysis, we excluded 3 HDIs that span centromeres on chromosomes 12, 16, and 18 as the size of these regions is likely overestimated due to the absence of typed SNPs and the very low recombination rate in these regions. Furthermore, we tested whether biological processes of the PANTHER gene ontology database [38,39] were enriched for genes in significantly differentiated regions.

SNPs were assigned to functional categories, such as genic and non-synonymous, based on information from ENSEMBL 54 [95]. First we tested if non-synonymous SNPs had a different *F_ST _*from other SNPs within HDIs and within LDIs using a Mann-Whitney *U *test. We then used a Fisher exact-test to investigate whether certain functional categories were enriched among SNPs in HDIs and LDIs. We additionally tested the enrichment of functional categories among SNPs identified in earlier steps of the analyses pipeline: i) the SNPs that were significant at the 1% level in the selection test, ii) the SNPs assigned to high-differentiation or low-differentiation states by the Viterbi algorithm, iii) and FDR-SNPs.

To test if non-genic islands are closer to genes than expected, we permuted non-genic HDIs and LDIs 1,000 times across the genome, conditioning on the fact that the permuted islands do not overlap with any gene. Using the same procedure, we also tested if non-genic islands overlapped more with transcription factor binding sites than expected by chance. To make this test, we used the list of transcription factor binding sites in the human genome available from ENCODE through the UCSC table browser [96,97]; table wgEncodeRegTfbsClustered].

### Comparison with previous studies

Previous genome scan studies have detected many candidate regions for both positive and balancing selection. We determined how many HDIs and LDIs overlapped with candidate regions discovered by other studies. We converted the positions of candidate regions from these studies into NCBI Build 36-reference system with the liftOver tool available on the UCSC web page if necessary [97]. For studies that identified genes instead of genomic regions we used the transcription start and transcription end of these genes as the limits of the candidate regions. Empirical *p*-values of the overlap with previous studies were obtained from 10,000 random permutations of HDI and LDI positions in the genome.

## Competing interests

The authors declare that they have no competing interests.

## Authors' contributions

TH developed the data analysis pipeline, performed statistical analyses, interpreted the results and wrote the manuscript. MF and LE were involved in project design, statistical analyses, and manuscript editing. All authors have read and approved the final manuscript.

## Supplementary Material

Additional file 1 **Comparison of observed and simulated *F_ST _*distributions**. Q-Q plots of observed *F_ST _*values and *F_ST _*values simulated under the FIM and the HIM. Click here for file

Additional file 2 **The steps involved in the HMM approach to detect HDIs and LDIs**. Illustration of the procedure used to identify islands with significant high and low population differentiation in the human genome. Click here for file

Additional file 3 **List of all detected HDIs and LDIs**. The properties of all HDIs and LDIs detected under the hierarchical island model. Click here for file

Additional file 4 **Test of enrichment of SNP categories in SNP subsets**. Fisher exact test for enrichment or depletion of SNPs with a given consequence to transcript (Ensembl) in genomic islands defined by the selection test or by the HMM approach. Click here for file

Additional file 5 **Island size and recombination rate**. Plots illustrating the relationship between island size and local recombination rate in genic and non-genic HDIs and LDIs. Click here for file

Additional file 6 **List of non-synonymous SNPs located in HDIs or LDIs with a *p*-value ≤ 0.05**. Candidate loci that might be affected by local adaptation or balancing selection, due to their effect on the transcript, significant level of population differentiation, and location within HDIs or LDIs, respectively. Click here for file

Additional file 7 **Overlap of HDIs and LDIs to candidate regions for selection identified in previous studies**. Histogram of recombination rate of all genomic bins, bins overlapping to HDIs, and bins overlapping to LDIs. Click here for file

Additional file 8 **Overlap of HDIs and LDIs to candidate regions for selection identified in previous studies**. Number of candidate regions for selection detected by previous studies that overlap with the 625 HDIs and 197 LDIs identified in this study [98,100-117]. Click here for file

Additional file 9 **Estimated parameters for the HMM**. Parameters of the HMM for each chromosome estimated by the Baum-Welch algorithm. (S8_HMM_parameters.xls can be viewed with Microsoft Excel or Excel Viewer). Click here for file
