# Supplementary material for: Evolutionary forces shaping genomic islands of population differentiation in humans
Source: BMC Genomics. 2012 Mar 22;13:107. doi: 10.1186/1471-2164-13-107 (PMC3317871; doi:10.1186/1471-2164-13-107)

## Additional file 1 – Comparison of observed and simulated *F_ST_* distributions

Q-Q plot of the observed *F_ST_* distribution and the *F_ST_* distribution simulated under the FIM (A, C) and the HIM (B, D) for chromosome 2 (A, B) and chromosome 21 (C, D). Observed and simulated *F_ST_* values were matched by their *H_BP_*. Note that the Q-Q plots of chromosomes 2 and 21 are representative for the other chromosomes.


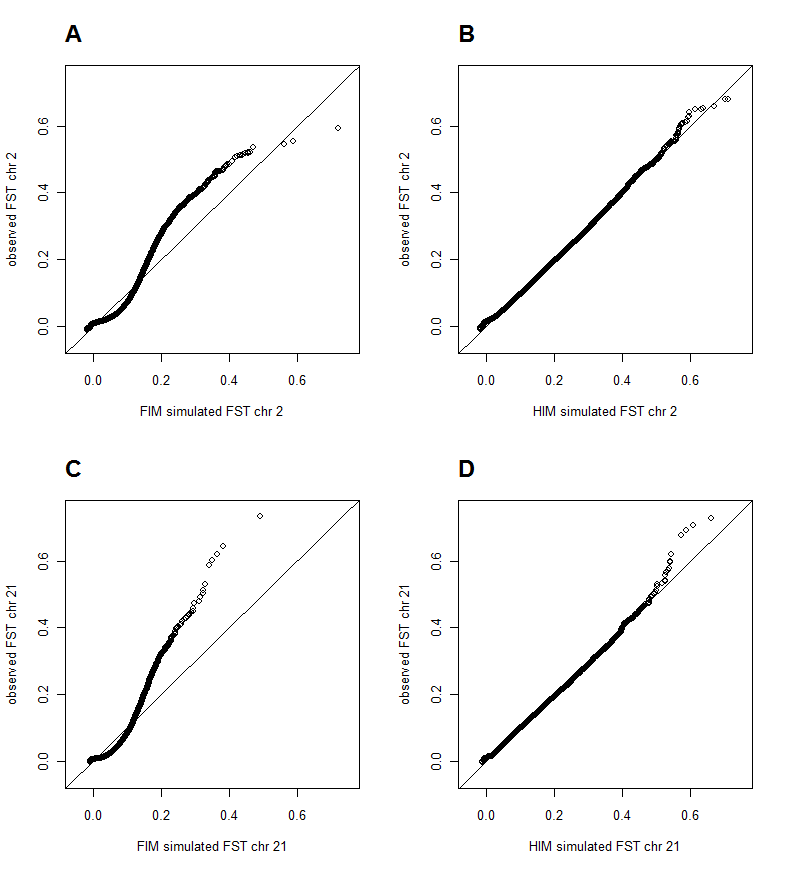

Supplement: Additional file 1 — Comparison of observed and simulated FST distributions. Q-Q plots of observed FST values and FST values simulated under the FIM and the HIM. [file 1471-2164-13-107-S1.DOC]
