# Supplementary material for: Evolutionary forces shaping genomic islands of population differentiation in humans
Source: BMC Genomics. 2012 Mar 22;13:107. doi: 10.1186/1471-2164-13-107 (PMC3317871; doi:10.1186/1471-2164-13-107)
Supplement: Additional file 2 — The steps involved in the HMM approach to detect HDIs and LDIs. Illustration of the procedure used to identify islands with significant high and low population differentiation in the human genome. [file 1471-2164-13-107-S2.DOC]

## Additional file 2 - The steps involved in the HMM approach to detect HDIs and LDIs

1. Input: A sequence of z-values ordered by chromosomal position.
2. Output from the Viterbi algorithm, leading to the most likely sequence of 3 possible chromosomal segment states: low-differentiation, neutral, and high differentiation.
3. Controlling for multiple testing: SNPs are assigned to either the high- or the low-differentiation state under a genome-wide False Discovery Rate (FDR) of 0.001, which is computed based on the probabilities of the SNPs to belong to each of the 3 states.
4. Identification of the genomic islands with the most significant low- or high-differentiation: Of the high- and low differentiation regions identified by the Viterbi algorithm (B) we only retain these that contain at least one FDR SNP as determined under step (C).


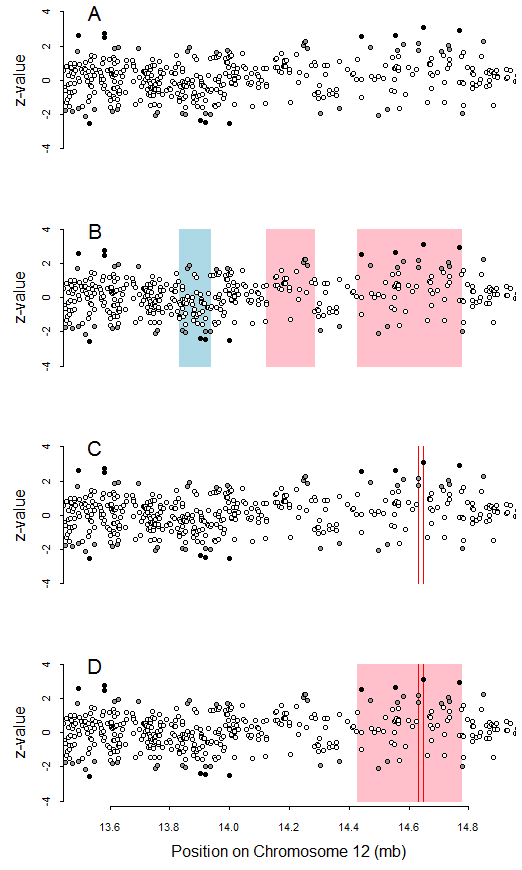


Legend
