# Supplementary material for: Evolutionary forces shaping genomic islands of population differentiation in humans
Source: BMC Genomics. 2012 Mar 22;13:107. doi: 10.1186/1471-2164-13-107 (PMC3317871; doi:10.1186/1471-2164-13-107)

## Additional file 5 – Association of island size and recombination rate

Relationship between size of genic and non-genic islands and their average recombination rate for HDIs (A) and LDIs (B).


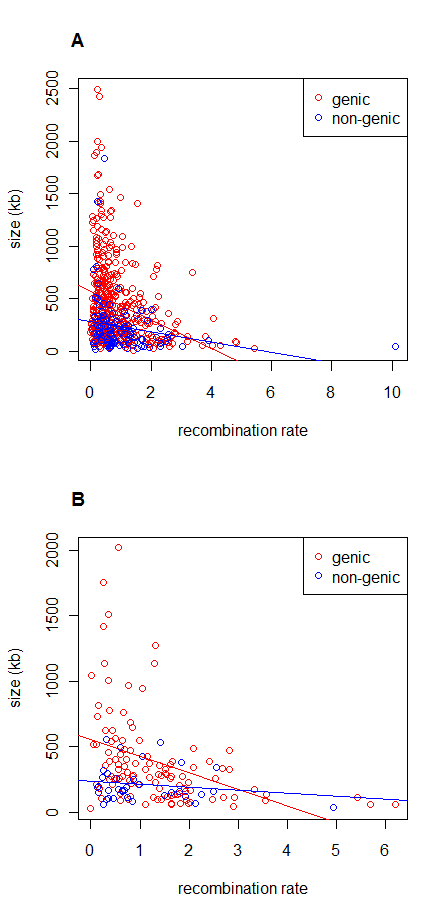

Supplement: Additional file 5 — Island size and recombination rate. Plots illustrating the relationship between island size and local recombination rate in genic and non-genic HDIs and LDIs. [file 1471-2164-13-107-S5.DOC]
