# Supplementary material for: Evolutionary forces shaping genomic islands of population differentiation in humans
Source: BMC Genomics. 2012 Mar 22;13:107. doi: 10.1186/1471-2164-13-107 (PMC3317871; doi:10.1186/1471-2164-13-107)

## Additional file 7 – Histogram of recombination rate

Histogram of relative sex-averaged DeCode recombination rate computed in bins of 10kb. Dark grey bars represent all genomic bins; medium grey bars represent bins overlapping with HDIs; light grey bars represent bins overlapping with LDIs.


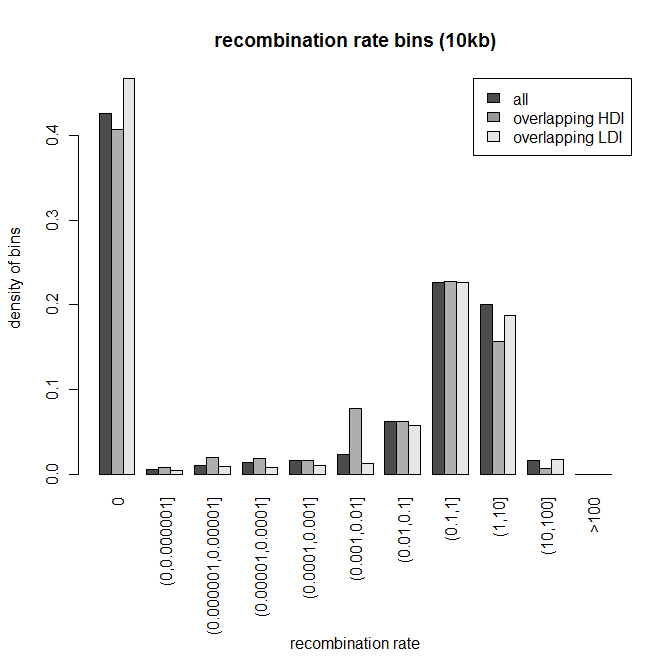

Supplement: Additional file 7 — Overlap of HDIs and LDIs to candidate regions for selection identified in previous studies. Histogram of recombination rate of all genomic bins, bins overlapping to HDIs, and bins overlapping to LDIs. [file 1471-2164-13-107-S7.DOC]
