# Supplementary material for: Evolutionary forces shaping genomic islands of population differentiation in humans
Source: BMC Genomics. 2012 Mar 22;13:107. doi: 10.1186/1471-2164-13-107 (PMC3317871; doi:10.1186/1471-2164-13-107)
Supplement: Additional file 8 — Overlap of HDIs and LDIs to candidate regions for selection identified in previous studies. Number of candidate regions for selection detected by previous studies that overlap with the 625 HDIs and 197 LDIs identified in this study [98,100-117]. [file 1471-2164-13-107-S8.DOC]

## Additional file 8 - Overlap of HDIs and LDIs to candidate regions for selection identified in previous studies

Number of candidate regions for selection detected by previous studies that overlap with the 625 HDIs and 197 LDIs identified in this study. Italic p-values indicate that there is less overlap than expected by chance.

|  | | | **No. of overlapping regions** | | | |
| --- | --- | --- | --- | --- | --- | --- |
| **Study** | **Method used to detect selection in other studies** | **Total No. of regions** | **Overlap with HDIs** | **p-value** | **Overlap with LDIs** | **p-value** |
| Sabeti et al. [1] positive selection | Candidate genes | 63 | 17 | 0.003 | 2 | 0.551 |
| Huttley et al. [2] | Linkage Disequilibrium | 10 | 7 | 0.433 | 2 | 0.485 |
| Wang et al. [3] | Linkage Disequilibrium | 87 | 14 | 0.344 | 3 | 0.446 |
| Voight et al. [4] | Linkage Disequilibrium | 714 | 141 | <0.001 | 12 | *0.020* |
| Frazer et al. [5] | Linkage Disequilibrium | 213 | 56 | <0.001 | 6 | 0.119 |
| Sabeti et al. [6] | Linkage Disequilibrium | 42 | 20 | <0.001 | 0 | 0.155 |
| Tang et al. [7] | Linkage Disequilibrium | 808 | 232 | <0.001 | 20 | *<0.001* |
| Pickrell et al. [8]  iHS | Linkage Disequilibrium | 1979 | 623 | <0.001 | 88 | 0.314 |
| Pickrell et al. [8]  XP-EHH | Linkage Disequilibrium | 1886 | 542 | <0.001 | 4 | *<0.001* |
| Carlson et al. [9] | Site Frequency Spectrum | 59 | 34 | <0.001 | 0 | *0.043* |
| Williamson et al. [10] | Site Frequency Spectrum | 180 | 74 | <0.001 | 2 | *0.046* |
| Hellmann et al. [11]  positive selection | Site Frequency Spectrum | 737 | 134 | <0.001 | 15 | *0.043* |
| Nielsen et al. [12] MWU-high | Site Frequency Spectrum | 524 | 67 | 0.209 | 15 | 0.244 |
| Nielsen et al. [12] MWU-low | Site Frequency Spectrum | 565 | 69 | 0.315 | 13 | 0.110 |
| Altshuler et al. [13] | Site Frequency Spectrum / Population differentiation | 19 | 11 | 0.003 | 0 | 0.167 |
| Nielsen et al. [12] G2D | Site Frequency Spectrum / Population differentiation | 287 | 28 | 0.251 | 7 | 0.195 |
| Chen et al. [14] | Site Frequency Spectrum / Population differentiation | 80 | 58 | <0.001 | 0 | *0.035* |
| Grossman et al. [15] | Linkage Disequilibrium / Population differentiation | 178 | 52 | <0.001 | 2 | *0.064* |
| Akey et al. [16]  positive selection | Population differentiation | 140 | 45 | <0.001 | 1 | *0.025* |
| Oleksyk et al. [17] | Population differentiation | 179 | 55 | <0.001 | 5 | 0.114 |
| Nielsen et al. [12]  F_ST_ | Population differentiation | 398 | 116 | <0.001 | 6 | *0.035* |
| Mikkelsen et al. [18] | Ratio of polymorphism to divergence | 15 | 5 | 0.432 | 0 | 0.161 |
| Bustamante et al. [19]  positive selection | Ratio of polymorphism to divergence | 294 | 47 | 0.015 | 6 | 0.119 |
| Green et al. [20] | Ratio of polymorphism to divergence | 212 | 42 | 0.036 | 4 | *0.050* |
| Sabeti et al. [1] balancing selection | Candidate genes | 21 | 2 | 0.613 | 0 | 0.396 |
| Hellmann et al. [11]  balancing selection | Site Frequency Spectrum | 1313 | 109 | *<0.001* | 29 | *0.038* |
| Akey et al. [16]  balancing selection | Population differentiation | 17 | 1 | 0.3725 | 0 | 0.512 |
| Andres et al. [21] | Ratio of polymorphism to divergence | 56 | 5 | 0.419 | 1 | 0.443 |
| Bustamante et al. [19]  balancing selection | Ratio of polymorphism to divergence | 776 | 84 | 0.330 | 19 | 0.109 |

# References

1. Sabeti PC, Reich DE, Higgins JM, Levine HZP, Richter DJ, Schaffner SF, Gabriel SB, Platko JV, Patterson NJ, McDonald GJ, et al: **Detecting recent positive selection in the human genome from haplotype structure.** *Nature* 2002, **419:**832-837.

2. Huttley GA, Smith MW, Carrington M, O'Brien SJ: **A scan for linkage disequilibrium across the human genome.** *Genetics* 1999, **152:**1711-1722.

3. Wang ET, Kodama G, Baidi P, Moyzis RK: **Global landscape of recent inferred Darwinian selection for Homo sapiens.** *Proceedings of the National Academy of Sciences of the United States of America* 2006, **103:**135-140.

4. Voight BF, Kudaravalli S, Wen X, Pritchard JK: **A map of recent positive selection in the human genome.** *PLoS Biology* 2006, **4:**e72.

5. Frazer KA, Ballinger DG, Cox DR, Hinds DA, Stuve LL, Gibbs RA, Belmont JW, Boudreau A, Hardenbol P, Leal SM, et al: **A second generation human haplotype map of over 3.1 million SNPs.** *Nature* 2007, **449:**851-U853.

6. Sabeti PC, Varilly P, Fry B, Lohmueller J, Hostetter E, Cotsapas C, Xie X, Byrne EH, McCarroll SA, Gaudet R, et al: **Genome-wide detection and characterization of positive selection in human populations.** *Nature* 2007, **449:**913.

7. Tang K, Thornton KR, Stoneking M: **A new approach for using genome scans to detect recent positive selection in the human genome.** *PLoS Biology* 2007, **5:**1587-1602.

8. Pickrell JK, Coop G, Novembre J, Kudaravalli S, Li JZ, Absher D, Srinivasan BS, Barsh GS, Myers RM, Feldman MW, Pritchard JK: **Signals of recent positive selection in a worldwide sample of human populations.** *Genome Research* 2009, **19:**826-837.

9. Carlson CS, Thomas DJ, Eberle MA, Swanson JE, Livingston RJ, Rieder MJ, Nickerson DA: **Genomic regions exhibiting positive selection identified from dense genotype data.** *Genome Research* 2005, **15:**1553-1565.

10. Williamson SH, Hubisz MJ, Clark AG, Payseur BA, Bustamante CD, Nielsen R: **Localizing recent adaptive evolution in the human genome.** *PLoS Genetics* 2007, **3:**e90.

11. Hellmann I, Mang Y, Gu ZP, Li P, de la Vega FM, Clark AG, Nielsen R: **Population genetic analysis of shotgun assemblies of genomic sequences from multiple individuals.** *Genome Research* 2008, **18:**1020-1029.

12. Nielsen R, Hubisz MJ, Hellmann I, Torgerson D, Andres AM, Albrechtsen A, Gutenkunst R, Adams MD, Cargill M, Boyko A, et al: **Darwinian and demographic forces affecting human protein coding genes.** *Genome Research* 2009, **19:**838-849.

13. Altshuler D, Brooks LD, Chakravarti A, Collins FS, Daly MJ, Donnelly P, Gibbs RA, Belmont JW, Boudreau A, Leal SM, et al: **A haplotype map of the human genome.** *Nature* 2005, **437:**1299-1320.

14. Chen H, Patterson N, Reich D: **Population differentiation as a test for selective sweeps.** *Genome Research* 2010, **20:**393-402.

15. Grossman SR, Shylakhter I, Karlsson EK, Byrne EH, Morales S, Frieden G, Hostetter E, Angelino E, Garber M, Zuk O, et al: **A composite of multiple signals distinguishes causal variants in regions of positive selection.** *Science* 2010, **327:**883-886.

16. Akey JM, Zhang G, Zhang K, Jin L, Shriver MD: **Interrogating a high-density SNP map for signatures of natural selection.** *Genome Research* 2002, **12:**1805-1814.

17. Oleksyk TK, Zhao K, De La Vega FM, Gilbert DA, O'Brien SJ, Smith MW: **Identifying selected regions from heterozygosity and divergence using a light-coverage genomic dataset from two human populations.** *PLoS One* 2008, **3**.

18. Mikkelsen TS, Hillier LW, Eichler EE, Zody MC, Jaffe DB, Yang SP, Enard W, Hellmann I, Lindblad-Toh K, Altheide TK, et al: **Initial sequence of the chimpanzee genome and comparison with the human genome.** *Nature* 2005, **437:**69-87.

19. Bustamante CD, Fledel-Alon A, Williamson S, Nielsen R, Hubisz MT, Glanowski S, Tanenbaum DM, White TJ, Sninsky JJ, Hernandez RD, et al: **Natural selection on protein-coding genes in the human genome.** *Nature* 2005, **437:**1153-1157.

20. Green RE, Krause J, Briggs AW, Maricic T, Stenzel U, Kircher M, Patterson N, Li H, Zhai WW, Fritz MHY, et al: **A draft sequence of the Neandertal genome.** *Science* 2010, **328:**710-722.

21. Andres AM, Hubisz MJ, Indap A, Torgerson DG, Degenhardt JD, Boyko AR, Gutenkunst RN, White TJ, Green ED, Bustamante CD, et al: **Targets of balancing selection in the human genome.** *Molecular Biology and Evolution* 2009, **26:**2755-2764.
